# Exploring Women’s Perceived Quality of Antenatal Care: A Cross-Sectional Study in The Netherlands

**DOI:** 10.3390/ijerph22091392

**Published:** 2025-09-06

**Authors:** Evelien Cellissen, Marijke Hendrix, Maaike Vogels-Broeke, Luc Budé, Marianne Nieuwenhuijze

**Affiliations:** 1 Research Centre for Midwifery Science, Zuyd University, Universiteitssingel 40, 6229 ER Maastricht, The Netherlands; marijke.hendrix@zuyd.nl (M.H.); luc.bude@zuyd.nl (L.B.); marianne.nieuwenhuijze@zuyd.nl (M.N.); 2 Care and Public Health Research Institute (CAPHRI), Maastricht University, 6200 MD Maastricht, The Netherlands; broeke@vogelsenbroeke.nl

**Keywords:** quality of care, continuity of care, antenatal care, woman-centered care, women’s experiences, coordinating care professional, maternity care plan

## Abstract

Evaluating antenatal care quality involves understanding women’s experiences and their impact on pregnancy outcomes. This study examines how pregnant women in the Netherlands perceive the quality of antenatal care and which factors are related to these perceptions, with a focus on continuity of care. We conducted a cross-sectional study (2019–2020) among 1165 pregnant women (>32 weeks). Perceived quality of care was measured using the Pregnancy and Childbirth Questionnaire. Experienced continuity of care was measured using the Nijmegen Continuity Questionnaire. Regression analyses explored associated factors across both community and hospital care settings. Most women reported moderate-to-high levels of perceived quality. Personal continuity from community midwives, team continuity, and the presence of a coordinating care professional were associated with higher perceived quality. The use of a maternity care plan showed no association. Our findings suggest that involvement of a community midwife enhances perceived quality of antenatal care. Key contributing factors include continuity of care and experiencing a coordinating care professional.

## 1. Introduction

A positive care experience in the perinatal period facilitates the transition to motherhood, enhances a woman’s sense of accomplishment and self-esteem, promotes the well-being of families, and influences future reproductive choices [1]. The World Health Organization and others recognize women’s experiences as an essential part of the quality of maternity care [2,3,4,5]. Positive experiences during antenatal care are associated with better preparation for birth and a more positive overall care experience, healthy pregnancy, and better perinatal outcomes [5,6,7,8]. To ensure a positive experience, maternity care should be organized around women’s needs, including understanding how women evaluate the quality of their maternity care [3,4,9,10,11].

Women value respectful and personalized care, access to clear and consistent information, and continuity of care as dimensions of quality maternity care [1,2,8,12,13,14,15,16,17]. Continuity of care is widely recognized as an important contributor to women’s care experiences [8]. Continuity is a multidimensional concept, referring to patients’ perception of how well their care is coordinated over time, with coherence and consistency across involved professionals, disciplines, and organizations [9,18,19]. Within this concept, three dimensions can be distinguished: informational continuity, referring to the consistent exchange of information between care professionals involved in the care for one person; managerial continuity, concerning the coordination and organization of care for one individual across professionals; and relational continuity, highlighting the ongoing relationship of trust between patients and care professionals [20,21,22]. Most research in this area focuses on relational continuity, especially in midwife continuity of care models, where women report more positive experiences when care is provided by a known professional or a small, consistent team [8,23,24,25]. The importance of understanding how different dimensions of continuity shape women’s perceptions of quality of antenatal care has been recognized, yet its relationship remains underexplored [13,26,27].

Furthermore, accessibility to midwife-led care varies across different countries, and often women with medium- or high-risk pregnancies do not receive midwife continuity of care [8,23]. This is also the case in the Netherlands, where the maternity care system is organized around a division between primary care in the community and secondary or tertiary care in hospitals [17,28]. Antenatal care is based on the assessment of each woman’s individual risk. Women with low-risk pregnancies receive care from community midwives in primary care settings led by midwives. Women with medium- or high-risk pregnancies receive care from obstetricians, residents, hospital-based midwives, and nurses, in secondary or tertiary care settings led by obstetricians, in this article further referred to as hospital staff. When complications arise during pregnancy in women initially considered low risk, they are referred from midwife-led to obstetrician-led care [29]. Despite attempts to implement midwife continuity of care models in the Netherlands [30], many barriers, such as a shortage of staff, make it currently hard to achieve. As a result, many women continue to be referred from community midwives to hospital staff and are therefore seen by multiple care professionals across different care settings. Since this fragmentation is thought to affect the quality of care, an integrated maternity care standard was introduced in 2016 to enhance continuity of care [31,32,33].

This care standard aims to establish a safe, woman-centered, and integrated maternity care system, requiring all Dutch maternity care professionals to implement its recommendations in practice. Key recommendations include a maternity care plan that outlines the agreements between the woman and care professional made for each phase of maternity care, incorporating women’s wishes and preferences [31,32], and a coordinating care professional, both intended to enhance continuity of care, improve alignment between care provision and women’s preferences, and ultimately improve the quality of maternity care.

Although the care standard was introduced in 2016, little is known about how women perceive quality of antenatal care within this system [17,34] and to what extent the two key recommendations from the integrated maternity care standard, maternity care plan, and coordinating care professional, contribute to women’s perceived quality of antenatal care. Therefore, our study explores the following:

– How pregnant women in the Netherlands perceive the quality of antenatal care;
– The association between perceived quality of antenatal care and experience of continuity during antenatal care and the key recommendations in the integrated maternity care standard: implementation of a maternity care plan and a coordinating care professional.

## 2. Materials and Methods

We used data from a cross-sectional study among pregnant women in the Netherlands, collected between February 2019 and February 2020. Our study is part of a larger cross-sectional study [StEM] [35] involving 3494 women during pregnancy (2nd and 3rd trimester) and postpartum. We analyzed the data of 1165 pregnant women in their 3rd trimester of pregnancy.

### 2.1. Participants

All midwifery practices and hospitals in the Netherlands were invited to participate. A total of 83 midwifery practices and 9 hospitals from different areas in the Netherlands volunteered and participated, reflecting both geographic diversity and the ratio of midwifery practices to hospitals in the Netherlands [36]. Care professionals invited pregnant women to participate, and additional recruitment was conducted via social media (Facebook and Twitter). Eligible participants had to be ≥32 weeks of pregnancy, >18 years old, and proficient in Dutch. Women experiencing perinatal death or severe neonatal morbidity in their current pregnancy were excluded.

### 2.2. Data Collection

All women who agreed to participate received a questionnaire by post or email. If necessary, two reminders were sent after one week and after three weeks. Before initiating the questionnaire, all respondents signed an informed consent form. The Medical Ethics Committee Z, Heerlen (registry number: METC-Z-20180121), approved the study.

The questionnaire contained questions about women’s background characteristics (age, educational level, parity, and nationality) and experienced number of care professionals with the response options: “very many”, “many”, “few”, and “very few”. We also asked women if they had a maternity care plan and a coordinating care professional, both with the response options: “yes”, “no”, and “Don’t know”. These last two questions were phrased in accordance with the Dutch integrated maternity care standard [31,32]. To explore women’s perceived quality of antenatal care, we used two subscales of the pregnancy section of the Pregnancy and Childbirth Questionnaire (PCQ) in our questionnaire: (1) ‘PCQ1-Personal treatment’ (11 items) and (2) ‘PCQ2-Educational information’ (7 items) [15]. The PCQ was designed especially to measure the quality of maternity care. We used the original subscale 1, and from subscale 2, we used 6 of the 7 original items. We excluded the item ‘information regarding normal delivery’, because it was not relevant to all participants at the time they completed the questionnaire. Participants scored the items on a five-point Likert scale (1 = strongly disagree to 5 = strongly agree), with higher scores indicating higher levels of perceived quality. To explore women’s experience of continuity during antenatal care, we used items of the Nijmegen Continuity Questionnaire (NCQ) containing three subscales: ‘Personal continuity: care professional knows me’ (NCQ1; 5 items), ‘Personal continuity: care professional shows commitment’ (NCQ2; 3 items), and ‘Team continuity: collaboration between care professionals’ (NCQ3; 4 items) [22,37]. Women who either received care from community midwives or from hospital staff throughout their pregnancy assessed their experience of continuity with the NCQ1, 2, 3. Women who were referred to receive care from both disciplines scored all three NCQ subscales twice, once for the experience of continuity from community midwives and once for hospital staff. Items were scored on a five-point Likert scale (1 = strongly disagree to 5 = strongly agree). A higher score indicates higher levels of experienced continuity of care [37]. In NCQ3, the “not applicable” option was available for women who received care from only one midwife.

### 2.3. Statistical Analyses

Items were phrased in positive and negative statements; scores for negative statements were reversed in alignment with the scores for positive statements. We present categorical variables as frequencies and percentages and continuous variables as means with standard deviations. Subscale scores for the PCQ and NCQ were calculated as the mean of the mean item scores within each subscale, resulting in scores ranging from 1 to 5 [15,37]. We analyzed three groups: women cared for only by community midwives, women cared for only by hospital staff, and women who were referred from community midwives to hospital staff due to a pregnancy complication. Differences in PCQ subscale means were analyzed using independent t-tests, comparing those who received care from midwives to those cared for by hospital staff. Additionally, differences were examined between two distinct groups: women who received care from community midwives and women who were referred during pregnancy. A *p*-value of ≤0.05 was considered statistically significant.

To address the responses “I don’t know” for the items “maternity care plan” and “coordinating care professional”, we recoded them as “no”, as women’s unawareness of these elements likely implied absence. To address the responses “not applicable” for the items on NCQ3, we recoded them to the midpoint of the Likert scale (score = 3, “neutral”). The item “experienced number of care professionals” was recoded by merging the response options “very many” and “many” into “many” and “few” and “very few” into “few”, resulting in a recoded dataset. Due to a design error in the questionnaire, some items from PCQ1 incorrectly included a “not applicable” response option, and the items for the NCQ incorrectly included a “I don’t know” response option. Therefore, we performed sensitivity analyses. The responses “not applicable” on the PCQ1 (14.9% of replies on the affected items) were replaced by the mean of the available cases. For the NCQ items, “I don’t know” responses (7.4% of replies on the affected items) were recoded to the midpoint of the Likert scale (score = 3, “neutral”). We compared these results with those from sensitivity analyses in which the cases with recoded variables were excluded. The results of the regression analyses based on this reduced dataset are provided in Appendix A. Analyses based on the recoded dataset are presented in the Section 3.

To explore factors associated with perceived quality of care, we performed two separate linear regression analyses. One model includes women who completed the NCQ for community midwives. This model shows the regression analyses for women who were cared for by community midwives as well as women who were referred and thereby received part of their care from community midwives. The second regression analysis included women who completed the NCQ for hospital staff. This model shows the analyses for women who were cared for by hospital staff and referred women receiving part of their care from hospital staff. This approach reflects the fact that participants completed the NCQ for each of these types of care professionals separately. Dependent variables were PCQ1 and PCQ2. Additional independent variables in the two regression analyses, besides the NCQ subscales for community midwives or hospital staff, were the maternity care plan and the coordinating care professional. Also added to our regression models were several potential confounding variables that were identified based on theoretical and empirical associations with perceived quality of care, including age, educational level, parity, nationality, number of care professionals involved, and referral status [8,25,38,39,40,41,42]. In our linear regression analyses, categorical variables with more than two levels were recoded into dummy variables, and cases with at least one missing value were labeled as system missing and excluded. Regression models were refined using a backward-selection strategy, removing non-significant variables iteratively until only significant predictors (*p* ≤ 0.05) remained. All analyses were performed using IBM SPSS Statistics, version 29.0 (IBM Corp., Armonk, NY, USA).

## 3. Results

**Figure 1:**
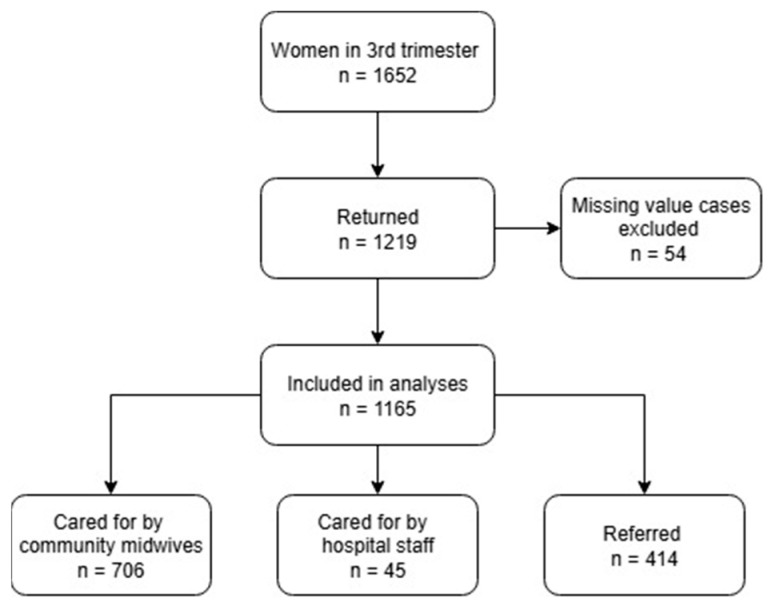
Response to the survey. Note: Women who were referred were cared for by both community midwives and hospital staff.

We distributed the questionnaire among 1652 women who were at least 32 weeks pregnant, and 1219 gave a response (response rate 73.8%). Because of missing values, we excluded 54 questionnaires from the analysis, leaving 1165 (70.5%) included responses. Among the participants, 706 women (60.6%) received exclusively primary care from community midwives, 45 women (3.9%) received exclusively secondary/tertiary care from hospital staff, and 414 women (35.5%) received both primary care from midwives and secondary/tertiary care from hospital staff (referred) (Figure 1).

Of all women, 264 women (22.7%) responded that they had a maternity care plan, and 502 women (43.1%) responded that they had a coordinating care professional (Table 1).

Among the group of women receiving care from community midwives and women who were referred, mean scores for continuity experienced from community midwives were 3.71 for personal continuity—‘care professional knows me’ (NCQ1), 3.66 for personal continuity—‘care professional shows commitment’ (NCQ2), and 4.04 for team continuity (NCQ3). The group of women receiving care from hospital staff and women who were referred reported mean scores of 2.83 (NCQ1), 2.87 (NCQ2), and 3.42 (NCQ3) for continuity of care received from hospital staff.

Differences in continuity of care experience during antenatal care between community midwives and hospital staff were statistically significant (*p* < 0.001). .

Table 2 presents the perceived quality of antenatal care with mean scores for the two subscales of the PCQ. Across all women, regardless of care professional or referral status, scores on personal treatment (PCQ1) were higher than those on educational information (PCQ2), with mean scores of 4.30 versus 3.87, respectively. Women receiving care from community midwives reported higher mean scores on both subscales compared to both women receiving care from hospital staff and women who were referred.

A in Table 3 presents the results of the first regression analysis examining the association between the independent variables and perceived quality of antenatal care (PCQ1: personal treatment; PCQ2: educational information) among women who received all or part of their care from community midwives. The independent variables included in this model were experience of continuity during antenatal care from community midwives, a coordinating care professional, a maternity care plan, and several confounding variables. In this model, experiencing continuity of care from community midwives (NCQ1 community midwives, NCQ2 community midwives, and NCQ3 community midwives) was associated with perceived quality of care (PCQ1 and PCQ2), except for team continuity (NCQ3), which was not associated with PCQ2. Experiencing a coordinating care professional and fewer care professionals contributed to higher levels of perceived quality of care (PCQ1). Being referred was associated with lower levels of perceived quality of care, both in personal treatment (PCQ1) and educational information (PCQ2). The performed sensitivity analyses for PCQ and NCQ confirmed that the main findings showed comparable results between the datasets, indicating that the results are robust irrespective of the recoding strategy applied (Appendix A). 

B in Table 3 shows the results of regression analyses examining the association between the independent variables and perceived quality of antenatal care (PCQ1: personal treatment; PCQ2: educational information) among women who received all or part of their antenatal care from hospital staff. The independent variables incorporated in the model were women’s experience of continuity of care as provided by hospital staff, a coordinating care professional, a maternity care plan, and several confounding variables. For personal treatment (PCQ1) and educational information (PCQ2), a positive association was found for team continuity from hospital staff (NCQ3 hospital staff). Experiencing a coordinated care professional and fewer care professionals were associated with higher levels of perceived quality of care on the two PCQ subscales. Being referred was associated with higher levels of personal treatment (PCQ1).

## 4. Discussion

### 4.1. Main Results

In this study, we explored how pregnant women in the Netherlands perceive the quality of antenatal care. Perceived quality was measured using the Pregnancy and Childbirth Questionnaire (PCQ) completed by pregnant women from 32 weeks of pregnancy onwards. Most women reported moderate-to-high levels of perceived quality of care, with the highest levels observed among women who received their care from community midwives, followed by women who were referred, and the lowest levels among women cared for by hospital staff. Additionally, we examined experienced continuity of care and the key recommendations in the integrated maternity care standard: implementation of a maternity care plan and a coordinating care professional, as potential factors associated with perceived quality of care. Experienced continuity of care from both community midwives and hospital staff was positively correlated with perceived quality of care. A coordinating care professional was associated with higher levels of perceived quality of care, whereas a maternity care plan was not.

### 4.2. Interpretation of Results

In our study, women reported a mean score of 4.30 for perceived quality of care—personal treatment (PCQ1) and 3.87 for perceived quality of care—educational information (PCQ2). These findings are consistent with previous studies reporting similar PCQ scores [15,34,43,44]. Although clinically meaningful cut-off scores on the PCQ subscales are undefined, a score of 3 corresponds to the neutral response category, and we interpret scores ≥ 3.5–5 as reflecting moderate-to-high perceived quality of care. In our study, we observed a trend in perceived quality of care, with the highest scores reported by women who received care from community midwives, followed by women who were referred, and the lowest scores reported by women who received care from hospital staff. In contrast with our findings, the earlier studies by Truijens et al. [15] and Lemmens et al. [34] reported the lowest perceived quality of care among women who were referred. A possible explanation for this difference is the timing of questionnaire completion. In the other studies [15,34], the questionnaire was administered postpartum, whereas in our study, women completed the questionnaire after 32 weeks of pregnancy. Therefore, it is possible that some women who were referred in our study had limited exposure to hospital staff, as most referrals occur late in the 3rd trimester [36]. This may indicate that their responses reflect the care received from community midwives, resulting in higher PCQ scores than in other studies [15,34]. This possible explanation may be supported by our descriptive analysis, which showed that women who received care from community midwives scored higher on the PCQ scales than both women who were referred and those who received care exclusively from hospital staff. Additionally, our regression analyses showed that referral was associated with perceived quality of antenatal care, but the direction of this association differed between the two regression models. In the model including women who received care from community midwives and women who were referred, the latter group experienced less ongoing involvement from community midwives compared to those who remained in community midwifery care throughout pregnancy. For these women, less involvement of community midwives was associated with lower perceived quality of antenatal care. In contrast, in the model including women who received care from hospital staff and women who were referred, referral indicates that women received antenatal care from a community midwife during at least part of their pregnancy, whereas women who were not received care exclusively from hospital staff. For these women, referral was associated with a higher perceived quality of antenatal care. These findings suggest that involvement of community midwives is positively associated with women’s perceived quality of antenatal care. They highlight the important role of community midwives and support existing recommendations that all women, regardless of risk status, should have access to care from community midwives during pregnancy. In case of medical risks, specialist care should be available in addition to care from community midwives [2]. This integrated approach is intended to promote positive care experiences and improve overall quality [8]. The specific mechanisms by which aspects of midwifery care contribute most to women’s perceived quality of care are still unknown. Qualitative research is needed to gain deeper insights into women’s perceptions and the mechanisms that influence those perceptions.

We explored additional factors contributing to variations in perceived quality of maternity care. Our regression analyses showed that experienced personal continuity of care from community midwives (NCQ1 and NCQ2) was positively associated with perceived quality of care. The importance of this personal continuity for women aligns with earlier research in which women consistently emphasized the value of being known and understood by a care professional they know and trust [8,38,45]. Personal continuity is mostly achieved within midwife continuity of care models, which have been shown to support stronger relationships between women and care professionals and lead to better outcomes [8]. Strengthening personal continuity through midwife-led care should be key to improving women’s perceived quality of antenatal care.

Our results also showed that for all women, experienced team continuity (NCQ3) was positively associated with perceived quality of care. Similar findings have been reported in other studies, which highlight the importance of seamless collaboration between professionals to ensure high quality of care [15,38,39]. However, achieving team continuity in practice remains challenging in the Dutch maternity care system [30]. Many women are referred from primary to secondary care during pregnancy [36], which often involves multiple care professionals and complicates the organization of care around the individual women. This increases the risk of care fragmentation and may result in perceived discontinuity of care. To support continuity of care in the Netherlands, the integrated maternity care standard includes key recommendations intended to enhance continuity and improve the quality of care [31,32]. Our study found that one of these recommendations, the presence of a coordinating care professional, was associated with higher perceived quality of antenatal care, while another, the use of a maternity care plan, showed no such association. While earlier studies suggest that both can positively influence women’s care experiences [18,46,47], the difference observed in our findings may be related to how these recommendations are used and experienced in practice. Coordinating care professionals are often actively involved throughout pregnancy, fostering collaboration and responding to women’s individual needs, elements that are likely to be noticed and valued by women [18,48]. Maternity care plans are often designed for and used to tailor care to women’s needs during birth and may not be perceived as relevant during the antenatal period [47,49]. It remains unclear if women perceive maternity care plans as helpful tools to improve continuity of care and whether professionals actively use them to improve continuity and personalize care. Understanding these mechanisms is crucial to ensure that these recommendations from the integrated maternity care standard (i.e., maternity care plan and coordinating care professional) are designed and implemented in ways that truly support continuity and enhance women’s perceived quality of care.

### 4.3. Strengths and Limitations

A strength of our study is the large, geographically diverse sample of 1165 pregnant women from across the Netherlands. Although our sample closely resembled the general pregnant population in the Netherlands, there was a slight overrepresentation of women with a Dutch nationality (89.8% vs. 86.3%) and women with a high level of education (58.5% vs. 53.7%) [36]. The exclusive availability of the questionnaire in Dutch will have excluded women with limited knowledge of the Dutch language. A strength of our study is the use of validated questionnaires that were administered during pregnancy, rather than postpartum, as is common in other studies. This timing reduces the risk of recall bias and allows for more accurate reporting of antenatal care experiences. Moreover, completing the questionnaires before birth ensures that women’s evaluations are not influenced by birth outcomes, which might affect their retrospective perceptions of care [50]. However, since the questionnaires were completed from 32 weeks of pregnancy onward, experiences occurring later in pregnancy may not have been fully captured. A limitation of our study is the small number of women who received care exclusively from hospital staff during pregnancy, which restricts our ability to draw detailed conclusions about this group.

## 5. Conclusions

Women in the Netherlands report moderate-to-high perceived quality of antenatal care. This study indicates that continuity of care plays an important role in shaping these perceptions. Perceived quality of antenatal care was positively associated with both personal continuity, particularly in the relationship with community midwives, and team continuity across care professionals. In addition, the involvement of a coordinating care professional was also linked to higher perceived quality of care. As the Dutch maternity care system develops toward more integrated models, it is essential to ensure that both collaboration between professionals and continuity in women’s care relationships are maintained. Since the involvement of community midwives is positively associated with women’s perceived quality of antenatal care across all risk groups, we recommend strengthening collaboration between professionals, with community midwives fulfilling a coordinating and continuous role in the care for pregnant women. Therefore, the role of community midwives should remain a priority within integrated care models to ensure high-quality, woman-centered care.

## Figures and Tables

**Table 1:** Characteristics.

Characteristics	Total (n = 1.165)	
	n	(%)
Age in years	Mean 30.5	
Educational level		
Low	56	(4.8)
Middle	427	(36.7)
High	682	(58.5)
Parity		
Nulliparous	439	(37.7)
Multiparous	726	(62.3)
Nationality		
Dutch	1035	(89.8)
Non-Dutch	130	(11.2)
Experienced a maternity care plan		
Yes	264	(22.7)
No	901	(77.3)
Experienced a coordinating care professional		
Yes	502	(43.1)
No	663	(56.9)
Experienced number of care professionals		
Many	426	(36.6)
Few	739	(63.4)
Referral status		
Unreferred		
Community midwives	706	(60.6)
Hospital staff	45	(3.9)
Referred	414	(35.5)

**Table 2:** Mean subscale scores of perceived quality of antenatal care measured with the Pregnancy and Childbirth Questionnaire (PCQ).

	Total (n = 1.165)		Community Midwives (n = 706)		Hospital Staff (n = 45)			Women Who Were Referred (n = 414)		
	Mean	(sd)	Mean	(sd)	Mean	(sd)		Mean	(sd)	
PCQ 1 Personal treatment	4.30	(0.47)	4.34	(0.44)	3.98	(0.54)	*p* < 0.001 *	4.25	(0.49)	*p* < 0.001 *
PCQ 2 Educational information during pregnancy	3.87	(0.62)	3.90	(0.62)	3.70	(0.64	*p* < 0.016 *	3.84	(0.62)	*p* < 0.045 *

* Women who received care from community midwives were the reference group.

**Table 3:** (**A**) Variables associated with women’s perceived quality of antenatal care (PCQ) among women who were not referred and received all of their care from community midwives and women who were referred and received care from both community midwives and hospital staff. (**B**) Variables associated with women’s perceived quality of antenatal care (PCQ) among women who were not referred and received all of their care from hospital staff and women who were referred and received care from both hospital staff and community midwives.

**A**								
	**PCQ1 (Personal Treatment)**				**PCQ2 (Educational Information)**			
	**Unstandard. β**	**Standard. β**	***p***	**CI**	**Unstandard. β**	**Standard. β**	***p***	**CI**
NCQ1_community midwives_	0.169	0.270	<0.001	[0.127–0.211]	0.205	0.245	<0.001	[0.146–0.263]
NCQ2_community midwives_	0.133	0.202	<0.001	[0.091–0.175]	0.221	0.251	<0.001	[0.159–0.283]
NCQ3_community midwives_	0.112	0.178	<0.001	[0.077–0.143]				
Parity					-	-	-	-
Nulliparous	Reference							
Multiparous	−0.049	−0.051	0.034	[−0.093–−0.004]				
Coordinating care professional	0.120	0.129	<0.001	[0.074–0.166]	-	-	-	-
Experienced number of care professionals								
Many	Reference				-	-		
Few	0.087	0.091	<0.001	[0.039–0.136]	-	-		
Referral status								
Unreferred	Reference				Referenc*e*			
Referred	−0.084	−0.088	<0.001	[−0.131–−0.038]	−0.079	−0.061	0.024	[−0.147–−0.011]
		R^2^ = 0.375				R^2^ = 0.206		
**B**								
	**PCQ1 (Personal Treatment)**				**PCQ2 (Educational Information)**			
	**Unstandard. β**	**Standard. β**	***p***	**CI**	**Unstandard. β**	**Standard. β**	***p***	**CI**
NCQ3 _hospital staff_	0.175	0.250	<0.001	[0.117–0.232]	0.168	0.193	<0.001	[0.093–0.243]
Coordinating care professional	0.181	0.181	<0.001	[0.097–0.264]	0.165	0.132	0.003	[0.556–0.273]
Experienced number of care professionals								
Many	Reference				Reference			
Few	0.249	0.249	<0.001	[0.165–0.332]	0.299	0.241	<0.001	[0.191–0.408]
Referral status								
Unreferred	Reference							
Referred	0.267	0.159	<0.001	[0.129–0.406]	-	-		
		R^2^ = 0.205				R^2^ = 0.126		

Note: (A) We removed variables that were not statistically significant from the model: maternity care plan, age, educational level, and nationality. (B) We removed variables that were not statistically significant from the model: personal continuity (NCQ1 and NCQ2), parity, maternity care plan, age, educational level, and nationality.

## Data Availability

The datasets presented in this article are not readily available due to technical limitations. Requests to access the datasets should be directed to the corresponding author.

## Appendix A

This appendix contains the results from the regression analyses on the reduced dataset.

**Table A1:** Variables associated with women’s perceived quality of antenatal care (PCQ) among women who received all or part of their care from community midwives—reduced dataset.

	PCQ1 (Personal Treatment)				PCQ2 (Educational Information)			
	Unstandard. β	Standard. β	*p*	CI	Unstandard. β	Standard. β	*p*	CI
NCQ1_community midwives_	0.109	0.162	<0.008	[0.028–0.190]	0.207	0.247	<0.001	[0.108–0.306]
NCQ2_community midwives_	0.145	0.212	<0.001	[0.071–0.219]	0.174	0.205	<0.001	[0.074–0.274]
NCQ3_community midwives_	0.192	0.270	<0.001	[0.114–0.270]				
Coordinating care professional	0.119	0.120	0.004	[0.039–0.198]	-	-	-	-
Referral status								
Unreferred	Reference				Reference			
Referred	−0.097	−0.098	<0.001	[−0.176–−0.019]	−0.079	−0.130	0.004	[−0.269–−0.051]
		R^2^ = 0.375				R^2^ = 0.190		

Note: This table presents results based on the reduced dataset and is part of the sensitivity analysis. It corresponds to A in Table 3 in the main text for comparison purposes.

**Table A2:** Variables associated with women’s perceived quality of antenatal care (PCQ) among women who received all or part of their care from hospital staff—reduced dataset.

	PCQ1 (Personal Treatment)				PCQ2 (Educational Information)			
	Unstandard. β	Standard. β	*p*	CI	Unstandard. β	Standard. β	*p*	CI
NCQ3_hospital staff_	0.211	0.329	<0.001	[0.131–0.291]	0.224	0.300	<0.001	[0.129–0.318]
Coordinating care professional	0.169	0.155	0.016	[0.032–0.305]	0.166	0.131	0.043	[0.556–0.273]
Experienced number of care professionals								
Many	Reference				Reference			
Few	0.242	0.218	<0.001	[0.103–0.381]	0.337	0.262	<0.001	[0.174–0.499]
Referral status								
Unreferred	Reference							
Referred	0.258	0.164	0.010	[0.062–0.454]	-	-		
		R^2^ = 0.232				R^2^ = 0.198		

Note: This table presents results based on the reduced dataset and is part of the sensitivity analysis. It corresponds to B in Table 3 in the main text for comparison purposes.
